# Institutional Notes and News

**Published:** 1911-06-10

**Authors:** 


### Institutional Notes and News.

**A Busy Cottage Hospital.**

The annual report of the Ashby-de-la-Zouch Cottage Hospital shows eighty-one admissions have taken place, with an average stay per patient of sixteen days. The district nurse has attended eighty-six cases and made upwards of three thousand visits. The balance in hand on the year's working was £58. Lady Maud Hastings and Mrs. F. Abel Smith have been elected vice-presidents. The maternity branch has rendered extensive service, and the Ladies' Working Association and the Sewing Party have been remarkably busy.

**The New Hospital at Malvern.**

Malvern, we learn, now possesses a new hospital in Lansdowne Crescent, on a site near the station, the gift of Mr. C. W. Dyson Perrins. It has accommodation for twenty-four beds, a theatre and an out-patient department. The Malvern district will be served by the new institution, which will take the place of the old hospital on Malvern Link, the proposed alterations to which on being found unpromising led, we believe, to Mr. Perrins' gift. The new hospital has been designed by Mr. William Henman, F.R.I.B.A., of Birmingham. Balconies are made a prominent feature of each large ward, and the hospital has a fine view and attractive grounds. The opening ceremony was performed by Lady Beauchamp.

**Innovations at Queen's Hospital for Children.**

Colonel Lord William Cecil, Chairman of the Committee of the Queen's Hospital for Children, Hackney Road, said last week at the annual meeting that he was glad they had been able to keep all the beds open; indeed, that the in-patients had increased by twenty-nine, and now amounted to 1,970. In the out-patients' department there had been 84,317 attendances, and the need for its enlargement was constantly being felt. They had, however, promised not to embark in any further building until the present mortgage debt was cleared off. Princess Henry of Battenburg, who visited the hospital last autumn, had kindly promised to visit it again on July 4 in connection with the Ladies' Association. The work of the lady almoner had continued to prove very successful not only in preventing abuse, but in looking after the children when they had left the hospital. He was sure they were all very sorry that Mr. Leonard Gibbs, the treasurer, had felt obliged to resign, but he was glad to think he would still be associated with them as a vice-president. Mr. Maurice Ruffer, an old friend, had accepted the place vacated by Mr. Gibbs, and on behalf of the Governors he wished to tender their thanks to Mr. Ruffer for again consenting to become their treasurer. There had been an innovation during the year in regard to the election of ladies on the committee, and it had been decided to appoint Lady W. Cecil and Miss Godlee. The festival dinner over which Lord Shaftesbury presided proved a fair success, and as a result they were able to place £1,780 to the mortgage redemption fund. The debt at the close of the year still stood at £6,200. The total gross expenditure had amounted to £14,404, and the total gross receipts to £15,018. King Edward's Fund had made them a grant of £3,000, of which £1,250 was earmarked for the reduction of the building debt. "The Little Folks" Convalescent Homes at Bexhill would enable them to discharge patients sooner than they could now. Princess Louise Duchess of Argyle will open the Home on July 13. The Home will contain thirty beds. The committee of the "Referee's" Children's Fund had given a donation of £2,000 for the purpose of endowing a ward in the Home, and they had a much larger project in view in the erection and maintenance of a separate convalescent establishment in connection with the hospital, which would be known as the "Referee" Children's Home. The Rev. J. E. Watts Ditchfield, in seconding, said the committee could not go on piling up deficits; 1910 began with one of £1,630 and ended up with a deficit of £3,671. By curing children and enabling them to grow up strong and healthy citizens the hospital was doing a work for the State, and was deserving of the support of all who had the welfare of the country at heart. The report was adopted. Mr. A. Clark, J.P., in proposing the election of the honorary officers and committee, remarked how pleased he was that it had been decided to include ladies on the committee.

**Insurance Bill and Middlesex Hospital.**

The following discussion at the quarterly Court of Governors of the Middlesex Hospital as to the probable effects of the Government's Insurance Bill is worth recording. Lord Sandhurst, who presided, was followed by Sir Alfred Pearce Gould, who said: It is quite clear that a very large proportion of patients who at present come to hospitals will, if the Bill becomes law, be provided for by the insurance scheme. If that be the case some of those who had been most generous to hospitals may reply that they had already responded to the appeal of the Chancellor of the Exchequer, and the hospital's funds may thus be very seriously affected. The question of the out-patients' departments ought also to be very carefully reconsidered. The efforts of hospitals were to some extent centred in lessening the number of out-patients who could more fairly be treated by their local practitioners. If the Bill becomes law the interests of the profession will be in the opposite direction. The State is proposing to give medical treatment, including drugs as well as sick pay, to practically all the wage-earning class of the community. Such illnesses as appendicitis or typhoid fever can be adequately dealt with only in hospitals, and the State will take up the position that it is to its interest that persons suffering from such diseases must be admitted to the hospitals. Without doubt that may lead on to a certain amount of State control of hospitals, which must be followed by their State maintenance.

